# Personalized Treatment Response in Progressive MS: Can the Patient's Profile Influence the Outcome?

**DOI:** 10.1002/brb3.70459

**Published:** 2025-06-10

**Authors:** Francesca Bovis, Ludwig Kappos, Sophie Arnould, Goeril Karlsson, Maria Pia Sormani

**Affiliations:** ^1^ Department of Health Sciences (DISSAL) University of Genova Genova Italy; ^2^ Research Center for Clinical Neuroimmunology and Neuroscience Basel (RC2NB), Departments of Head, Spine and Neuromedicine, Clinical Research, Biomedicine and Biomedical Engineering University Hospital and University of Basel Basel Switzerland; ^3^ Novartis Pharma AG Basel Switzerland; ^4^ IRCCS Policlinico San Martino Hospital Genoa Italy

**Keywords:** clinical trials, multiple sclerosis, personalized medicine, personalized treatment effects

## Abstract

**Background:**

Evidence from clinical trials providing average effects in populations is often used to forecast individualized patient outcomes similar to the trial patients. Multiple sclerosis (MS), known for notable heterogeneity in outcomes, makes the evaluation of potential heterogeneity of treatment effect (HTE) significant. Identifying factors that predict individual treatment response is crucial for optimizing patient care, and this study aimed to demonstrate the feasibility (proof of concept) of applying a statistical method to predict individual treatment response in MS trials.

**Methods:**

We developed an individualized response score (RS) to predict treatment response in patients with active secondary progressive MS (SPMS). The RS was a continuous combination of baseline clinical characteristics, including age, sex, previous relapses, EDSS, and disease duration. We used data from the EXPAND trial to train and validate the RS. A training dataset (70% of the data) was used to identify optimal response thresholds for four key outcomes: Expanded Disability Status Scale (EDSS), Timed 25 Foot Walk (T25FW), 9‐Hole Peg Test (9HP), and the Symbol Digit Modalities Test (SDMT). The remaining 30% of the data served as a validation set to assess the RS's predictive performance. The continuous RS was binarized (into responder and non‐responder) based on the threshold representing the top 25% versus the bottom 75% of the continuous score distribution.

**Results:**

Using baseline profiles, SPMS patients exhibiting varying benefits from Siponimod across different outcomes were successfully categorized as responders or non‐responders. The overall effect of Siponimod on the EDSS was HR = 0.79 (95% CI: 0.65‐0.95), while responders’ demonstrated a HR = 0.64 (95% CI: 0.49‐0.84) versus a HR = 0.97 (95% CI: 0.74‐1.27) for non‐responders’, interaction p = 0.027. Siponimod's overall effect on SDMT progression was HR = 0.75 (95% CI: 0.63‐0.88). Responders' demonstrated a HR = 0.59 (95% CI: 0.43‐0.80) vs a HR = 1.00 (95% CI: 0.69‐1.44) for non‐responders, interaction p = 0.031. On the entire dataset, Siponimod exhibited a non‐significant effect on 9HPT (HR = 0.86, 95% CI: 0.66‐1.10) and on T25FW (HR = 0.95, 95% CI: 0.81‐1.12), whereas responders’ demonstrated a HR = 0.68 (95% CI: 0.47‐0.97) on 9HPT and a HR = 0.77 (95% CI: 0.60‐0.98) for T25FW.

**Conclusions:**

This analysis demonstrated the ability to define responders to a therapy based on their baseline profile and evaluate the treatment effect on multiple endpoints, showing that the benefit on different outcomes can vary across patients.

## Graphical abstract

Multiple sclerosis (MS) is a highly heterogeneous disease, making it challenging to predict individual treatment responses. This study explored the feasibility of a statistical approach to identify patients most likely to benefit from treatment in clinical trials. Using data from the EXPAND trial, we developed an individualized response score based on baseline characteristics to predict Siponimod's effect in secondary progressive MS. Patients were categorized as responders or non‐responders, revealing significant variations in treatment outcomes across key measures. These findings highlight the potential for personalized treatment strategies, allowing for more tailored and effective therapy selection in MS.

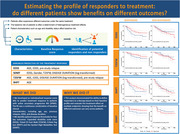

## 1 Introduction

Developments in the treatment of Relapsing multiple sclerosis (RMS) have resulted in a broad spectrum of therapies, predominantly anti‐inflammatory and immunomodulatory, for individuals with relapsing forms of the disease. Despite this success, many of these therapies have minimal impact on the progressive forms of Multiple sclerosis (MS); thus fewer treatment options are available to this patient cohort. However, positive effects seen in recent phase 3 immunomodulatory therapy trials for progressive MS (Montalban et al. 2017; Kappos et al. 2018) appear promising. Recent meta‐analyses highlight the importance that baseline demographics and disease characteristics have on the extent to which active inflammation influences trial outcomes. (Capanna et al. 2022)

The primary outcome of clinical trials in progressive MS usually features the Expanded Disability Scale Score (EDSS). (Kurtzke 1983) The limitation of EDSS to accurately capture other complex functional domains of MS disability, such as arm/hand dexterity and cognitive impairment, is known (Whitaker et al. 1995; van Munster and Uitdehaag 2017) and led to the development of the quantitative performance measures, including the Timed 25 Foot Walk (T25FW), 9‐Hole Peg Test (9HPT), and Paced Auditory Serial Addition Test (PASAT). Further research subsequently recommended the adoption of the Symbol Digit Modalities Test (SDMT). (Cutter et al. 1999; Rudick et al. 1997; Miller et al. 2000; Rudick et al. 2001)

Assessing heterogeneity of treatment effects (HTE), which captures variations in treatment response among different patient populations, is a fundamental aspect of precision medicine, allowing individualized treatment plans based on patient‐specific characteristics. (Kent et al. 2018; Sormani et al. 2023) Predictive modeling, which involves using statistical and machine learning techniques to forecast outcomes based on multiple patient attributes, plays a crucial role in identifying these variations. While the predominant HTE assessment tool is a one‐variable‐at‐a‐time subgroup analysis, predictive simultaneous multi‐characteristic modeling approaches are central to personalized predictive evidence‐based precision medicine (Kent et al. 2018; Sormani et al. 2023) as recently applied to MS. (Pellegrini et al. 2020; Bovis et al. 2019; Bovis et al. 2021; Falet et al. 2022) This study introduces an innovative application of predictive HTE modeling in Secondary Progressive MS (SPMS) by developing a continuous response score that integrates multiple baseline covariates to stratify patients based on their likelihood of treatment benefit. It serves as a proof‐of‐concept, demonstrating the feasibility of defining responder and non‐responder profiles in SPMS patients—a critical step toward more tailored therapeutic decision‐making.

This analysis applies predictive HTE together with a more personalized understanding of individual treatment outcomes by examining the treatment effect on multiple endpoints. By examining the patient benefit variations on a range of different outcomes, it is possible to better evaluate how the effectiveness of treatments for specific patient populations can be evaluated and determine the most likely patients to benefit from a particular therapy.

## 2 Materials and Methods

Here, we create a “response score” (RS), a linear combination of baseline factors that correlates with the size of each patient's expected treatment benefit. Post‐hoc analysis was performed on data from the EXPAND study, a randomized controlled trial of SPMS patients who received Siponimod or placebo. The study design details have been reported elsewhere. (Kappos et al. 2018) In brief, the inclusion criteria were aged 18–60 years, a diagnosis of SPMS (Rovaris et al. 2006) with an EDSS score of 3.0–6.5 at screening, a history of RRMS (2010 McDonald criteria), (Polman et al. 2011) evidence of EDSS progression in the 2 years prior to the study, and no signs of relapse in the previous three months before randomization.

### 2.1 Definitions of Outcomes

In order to identify specific patient subgroups based on their baseline characteristics that exhibit a greater benefit from the treatment, we must define the “benefit” of the treatment.

The treatment effect is defined as the ability of treatment to delay progression on the following four clinical outcomes: EDSS, T25FW, 9HPT, and SDMT as follows:

‐ EDSS progression is defined as a 1‐point increase in EDSS if the baseline score was 3.0–5.0, or a 0.5‐point increase if the baseline score was 5.5–6.5, confirmed at a scheduled visit at least three months later.
‐ T25FW progression and 9HPT progression, defined as worsening by at least 20% from baseline confirmed at a scheduled visit at least three months later.
‐ SDMT progression, defined as 6‐month confirmed worsening by ≥ 4 points of the SDMT score.

### 2.2 Testing and Validating Procedure

Training and validation datasets can be sampled from the same homogeneous population. The EXPAND trial dataset was split randomly, 70%:30%, into single training and validation sets. The RS for each outcome was defined using the training set and validated using the validation sample. The demographic and clinical characteristics and the outcomes between the two cohorts were compared using Cohen's d values to express standardized mean or proportion differences. Absolute values of *d* > 0.10 were considered clinically meaningful (Supplementary Material Table S1). A final model was then recalculated on the whole EXPAND dataset (Supplementary Material Table S5).

### 2.3 Baseline Variable Grouping

A specific set of baseline variables from the data collected in the EXPAND trial were used as potential components of the RS. These variables was used to create a linear combination that is associated with the magnitude of the treatment effect.

We defined four different subgroups of baseline variables:

1. The CLINICAL PRACTICE subgroup included only clinical variables routinely collected at baseline in clinical practice: age, sex, relapses in the previous 2 years, disease duration (log‐transformed), and EDSS score.
2. The second (ADVANCED CLINICAL) subgroup included clinical information on the 9HPT, the T25FW, and the SDMT scores in addition to the CLINICAL PRACTICE variables.
3. The third (CLINICAL and MRI) subgroup added the MRI variables routinely collected at baseline (Gd+ lesion count and T2 lesion volume) to the ADVANCED CLINICAL variables.
4. The fourth (EXPERIMENTAL) subgroup added a CLINICAL and MRI subgroup to the biomarkers used at a research level (normalized cortical grey matter volume, normalized thalamus volume, Glial Fibrillary Acidic Protein (GFAP, log‐transformed) and Neuro‐filament Light (NFL, log‐transformed), blood concentration).

We generated an RS for each outcome and set of baseline variables using the training set as outlined in previous studies (Pellegrini et al. 2020; Bovis et al. 2019, 2021, 2019, 2021; Falet et al. 2022; Zhao et al. 2013) and further detailed in the Supplementary Appendix‐A.

The validity of the RS was assessed on the validation set. Here, we present the results for the CLINICAL PRACTICE subgroup, with the findings of all other subgroups reported in Supplementary Appendix‐B. The CLINICAL PRACTICE subgroup was selected for emphasis due to its clinical relevance and its potential to directly inform clinical practice guidelines. While other subgroups contribute valuable insights into the underlying disease biology, their immediate translational value in routine clinical settings may be less pronounced.

### 2.4 Procedure for Selection of the Response Score Model

The RS was built using all 2^n^‐1 possible combinations of n baseline variables of the selected training set as previously described (Bovis et al. 2019) and often referred to as the “all possible‐subset regression” approach. This procedure assumes that at least one response score will evidence heterogeneity of treatment effect, increasing the likelihood of selecting the model that optimally balances goodness of fit and complexity.

To briefly illustrate how the HTE analysis works, let's suppose to have three baseline variables—age, EDSS, and sex—In this case, seven possible models would be generated: one for each individual variable (age, EDSS, sex) and four combining these variables (age+sex, age+EDSS, EDSS+sex, and age+sex+EDSS). According to the predictive modeling procedure for HTE analysis, two separate prognostic models were developed: one for the control group and one for the treatment group. If no treatment effect modification according to baseline variables is present, the coefficients of the two models are expected to be similar, reflecting identical prognostic predictions in both groups. Conversely, if the treatment has a different effect according to the levels of the baseline variables, the coefficients in the two models will result to be different. The score predicting treatment response is then derived from the difference of the coefficients between these two models and is a continuous score. To identify patients with heterogeneous response to treatment, for each RS, we arbitrarily pre‐defined a cut‐off at the first quartile of its distribution; that is, we categorized the scores into responder and non‐responder groups based on the threshold, representing the top 25% versus the bottom 75% of the continuous score distribution.

All the resultant RS were ranked in ascending order, according to their ability to discriminate potential responders and non‐responders. This property was defined as a ratio between the treatment effect (expressed as a hazard ratio (HR)) in the two groups (responders versus non‐responders). The model with the lowest HR (indicating the largest between‐group difference treatment effect) was then tested on the validation set, and the RS was considered “validated” if the HR was < 0.80 (indicating that the treatment effect was ≥ 20% higher in the responders group) and/or the p for treatment by score interaction was < 0.20 (since a treatment by subgroup interaction is a low‐power test, a liberal *p* value of 0.20 was considered a sufficient interaction indicator). In Appendix A, a detailed example demonstrating the specific steps involved in selecting the best model was presented.

This generated an RS for the four progression outcomes, each containing a specific set of baseline variables. Each patient‐specific RS is a continuous score representing the expected treatment effect (the expected HR on a log scale). Thus, the treatment effect in potential responders and non‐responders was defined according to an HR threshold equal to the HR on the whole group.

Individuals were classified and defined as “potential” responders or non‐responders using their baseline characteristics. Consequently, even placebo‐treated patients are categorized as potential responders or non‐responders.

### 2.5 Handling of Missing Data for Baseline Variables

We used multiple imputation by chained equations (Hayati Rezvan et al. 2015) (10 imputations) to impute missing values under an assumption of missingness at random, modeling EXPAND trial variables used as components of the RS.

### 2.6 Performance Evaluation

Possible measures to assess the performance of prediction models, including HTE models, include

- Measures of *discrimination*, i.e., a quantification to what extent the models can discriminate between patients who benefit and those who don't
- Measures of *agreement* between predicted and observed values
- Measures of how well selection of a given model could be *replicated*

Measures of discrimination and of agreement were evaluated in the validation set (30% of the overall EXPAND dataset). The rate of replication was derived by bootstrapping 500 training/validation samples from the overall EXPAND dataset. All these performance measures were detailed in Appendix A.

### 2.7 Variable Importance

We employed a conditional random forest variable importance analysis to identify the components of the RS that carried greater significance in the identification process to distinguish between responders and non‐responders.

## 3 Results

This analysis featured 1,645 patients, 1,099 in the Siponimod group and 546 in the placebo group.

Table 1 reports the effect of Siponimod versus placebo on the four progression outcomes. On the whole enrolled cohort, there was a significant effect on EDSS (HR = 0.79, *p* = 0.013) and on SDMT (HR = 0.75, *p* = 0.001), a modest and non‐significant effect on 9HPT (HR = 0.86, *p* = 0.23), and an HR = 0.95, *p* = 0.53 on T25FW progression.

**TABLE 1:** Treatment effect on the four confirmed disability progression outcomes in the EXPAND trial.

Outcome	HR	95%CI	*p* value
**EDSS**	0.79	0.65‐0.95	0.013
**9HPT**	0.86	0.66‐1.10	0.23
**T25FW**	0.95	0.81‐1.12	0.53
**SDMT**	0.75	0.63‐0.88	0.001

Abbreviations: 9HPT: 9‐hole peg test; CI = confidence Interval; EDSS: expanded disability status scale; HR: hazard ratio; SDMT: symbol digit modalities test;T25FW: timed 25‐foot walk test.

The whole population was randomly split into training (N = 1,152) and validation sets (N = 493).

Table 2 presents the RS composed of a linear combination of the CLINICAL PRACTICE subgroup of variables for each clinical outcome, including replicability metrics associated with each RS. The rate of replication of the RS was 71% for the EDSS, 73% for the 9HPT, 70% for the T25FW, and 76% for the SDMT.

**TABLE 2:** Response Score obtained on the training set using the CLINICAL PRACTICE subgroup of variables (age, sex, relapses in the previous 2 years, disease duration (log‐transformed) and EDSS score). The performance (Replicability rate and p for treatment by RS interaction were obtained on the validation set).

Outcome	Replicability rate (%)**	Response Score*	*p* for treatment by RS interaction**	AUC
EDSS	**71%**	−1.23 + 0.01 x age + 0.08 x EDSS–0.05×2y prior relapses	0.144	0.403
9HPT	**73%**	−0.39 + 0.005 x age	0.078	0.587
T25FW	**70%**	−0.58 + 0.01 x age + 0.11 x EDSS–0.19 x log(disease duration)–0.09×2y prior relapses	0.077	0.485
SDMT	**76%**	−0.56 + 0.10 x EDSS + 0.34 x male gender–0.15 x log(disease duration)	0.022	0.536

*Obtained on the training set (N = 1152) **Obtained on the validation set (N = 493).

Abbreviations: 9HPT: 9‐hole peg test; AUC: area under the AD(q) curve represents the curve generated by plotting the cumulative distribution of patients ranked by individual treatment response score and the overall treatment effect relative to a given proportion of patients. The lower is the curve, the higher the heterogeneity of treatment effect; EDSS: expanded disability status scale; SDMT: symbol digit modalities test; T25FW: timed 25‐foot walk test.

**FIGURE 1:**
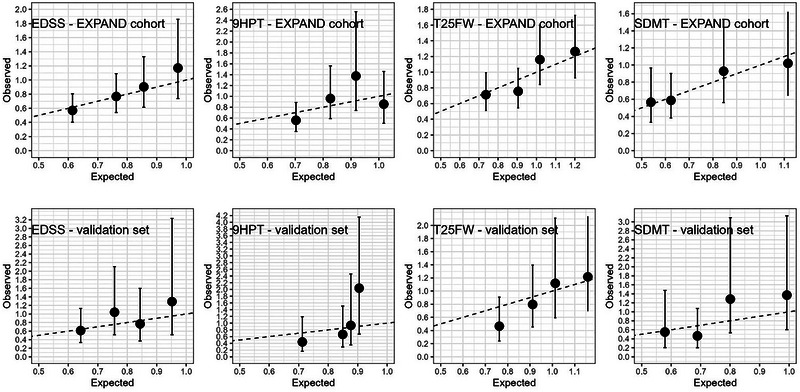
Calibration plot of the Response Score obtained on the CLINICAL PRACTICE subgroup of baseline variables (EDSS, 9HPT, T25FW, and SDMT) on the validation set (N = 493) and on the whole population (N = 1,645). 9HPT, 9‐hole peg test; EDSS, expanded disability status scale; SDMT, symbol digit modalities test; T25FW, timed 25‐foot walk test.

The RS calibration curves for the validation and whole population sets are reported in Figure 1. The RS quartile‐defined subgroup curves show the observed treatment effects (dots, representing the log (HR) with 95% CI on each subgroup) are consistent with the effect predicted by the RS (solid line), although the wide confidence limits indicate a large variability.

### 3.1 Response Score on EDSS Progression

The RS for EDSS progression has the following formulation:

$$\begin{array}{ccc} & & RS_{\mathrm{EDSS}}=-1.23+0.01\times \mathrm{age}+0.08\;\\ & & \times \mathrm{EDSS}-0.05\times 2-\mathrm{year}\;\mathrm{prior}\;\mathrm{relapses} \end{array}$$

The score is interpreted as a patient‐specific log (HR), (the treatment effect size on a log scale), where negative coefficients indicate larger benefits. The treatment by RS_EDSS_ interaction (*p* = 0.144) indicates the presence of heterogeneity in the treatment effect according to different levels of the RS, as per the pre‐specified criteria.

**FIGURE 2:**
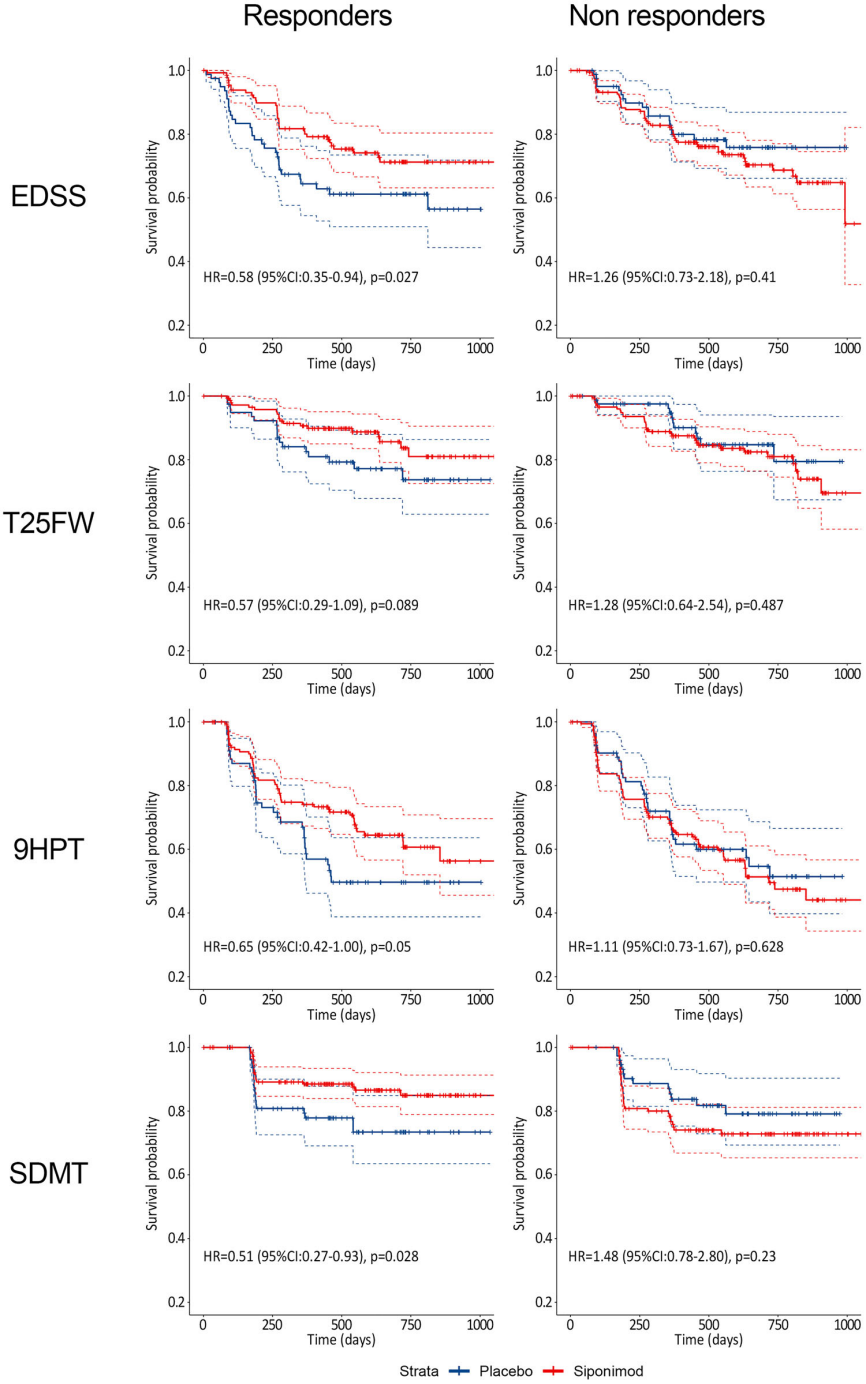
Kaplan–Meier survival curves for the progression free survival on EDSS, 9HPT, T25FW and SDMT, in responders and non‐responders as defined by the response score (Validation set, N = 493). 9HPT, 9‐hole peg test; CI, confidence interval; EDSS, expanded disability status scale; HR, hazard ration; SDMT, symbol digit modalities test; T25FW, timed 25‐foot walk test.

Table 3 and Figure 2 report the RS responder, non‐responder discrimination ability. The HR for EDSS progression on the whole population for Siponimod versus placebo was 0.79. Potential responders were thus classified if their exp(RS) was ≤ 0.79 and non‐responders if their exp(RS) was > 0.79. The observed treatment effect of potential responders was large (HR = 0.58, 95% CI: 0.35‐0.94) and significantly different to potential non‐responders (HR = 1.26, 95% CI: 0.73‐2.18), interaction *p* = 0.029).

**TABLE 3:** Treatment effect on progression outcomes by response score groups in the validation set (N = 493).

	RS cut‐off	Score group	Number of patients		Treatment effect	
			Placebo	Siponimod	HR (95% CI)	*p* ^*^
**EDSS**	log(0.79) = ‐0.24	Potential responders N = 213 N = 412	79	134	**0.58 (0.35‐0.94)**	0.029
		Potential non‐responders N = 280 N = 1233	82	198	**1.26 (0.73‐2.18)**	
**9HPT**	log(0.86) = ‐0.15	Potential responders N = 226 N = 412	78	148	**0.57 (0.29‐1.09)**	0.075
		Potential non‐responders N = 267 N = 1233	83	184	**1.28 (0.64‐2.54)**	
**T25FW**	log(0.95) = ‐0.05	Potential responders N = 236 N = 413	79	157	**0.65 (0.42‐1.00)**	0.079
		Potential non‐responders N = 257	82	175	**1.11 (0.73‐1.67)**	
**SDMT**	log(0.75) = ‐0.27	Potential responders N = 277	82	195	**0.51 (0.27‐0.93)**	0.015
		Potential non‐responders N = 216	79	137	**1.48 (0.78‐2.80)**	

Abbreviations: 9HPT: 9‐hole peg test;EDSS: expanded disability status scale; RS: response score; SDMT: symbol digit modalities test; T25FW: timed 25‐foot walk test.

* p for treatment by score interaction.

Table 4 details the variable importance ranking and shows the most important treatment effect modifier on EDSS progression was younger age, followed by a lower baseline EDSS score (implying less clinical disability).

**TABLE 4:** Variables predicting treatment response score in the validation set (N = 493), ranked in order of importance using the CLINICAL PRACTICE set of variables.

	Variables included in the Response Score	VI^*^	%VI
**EDSS**	Age	0.017	100
	EDSS	0.012	73
	No. 2‐year prior relapses	0.003	17
**9HPT**	Age	0.004	100
**T25FW**	EDSS	0.018	100
	log(disease duration)	0.011	61
	No. 2‐year prior relapses	0.007	42
	Age	0.003	18
**SDMT**	Male sex	0.045	100
	EDSS	0.017	39
	Log (disease duration)	0.015	34

Abbreviations: 9HPT: 9‐Hole Peg Test;EDSS: expanded disability status scale; SDMT: symbol digit modalities test; T25FW: timed 25‐foot walk test.

* VI: Variable importance derived from random forest conditional variable importance measures.

### 3.2 Response Score on 9HPT Progression

The RS for 9HPT has the following formulation:

$$\;RS_{9\mathrm{HPT}}=-0.39+0.005\times \mathrm{age}$$

The treatment by RS_9HPT_ interaction (*p* = 0.078) indicates the heterogeneity of treatment effect on the 9HPT progression according to different levels of the RS. The treatment effect on 9HPT progression in potential responders and non‐responders is reported in Table 3 and in Figure 2. In the potential responder group, (46% of the validation set) the treatment effect was large (HR = 0.57, 95% CI: 0.29‐1.09) versus a HR = 1.28, 95% CI: 0.64‐2.54) for potential non‐responders group (interaction *p* = 0.075). The only driver of response on this outcome is a younger age.

### 3.3 Response Score on T25FW Progression

The RS for T25FW (RS_T25FW_) has the following formulation:

$$\begin{array}{ccc} & & \;RS_{T25\mathrm{FW}}=-0.58+0.01\;\times \mathrm{age}+0.11\times \mathrm{EDSS}-0.19\\ & & \times \log\left(\mathrm{disease}\;\mathrm{duration}\right)-0.09\times 2-\mathrm{year}\;\mathrm{prior}\;\mathrm{relapses} \end{array}$$

The treatment by RS_T25FW_ interaction (*p* = 0.077) indicates the heterogeneity of treatment effect on the T25FW progression according to different levels of the RS. The treatment effect on T25FW progression in potential responders and non‐responders reported in Table 3 and in Figure 2. In the potential responders group (48% of the validation sample) the treatment effect was larger (HR = 0.65, 95% CI: 0.42‐1.00) than the potential non‐responder group (HR = 1.11, 95%CI:0.73‐1.67) (interaction *p* = 0.079).

The variable importance ranking reported in Table 4 indicated that the most important driver of response to treatment on T25FW progression is a lower EDSS score and a shorter disease duration.

### 3.4 Response Score on SDMT Progression

The RS for SDMT (RS_SDMT_) has the following formulation:

$$\begin{array}{ccc} & & \;RS_{\mathrm{SDMT}}=-0.56+0.10\;\times \mathrm{EDSS}+0.34\;\times \mathrm{male}\;\mathrm{sex}-0.19\;\\ & & \times \log\left(\mathrm{disease}\;\mathrm{duration}\right) \end{array}$$

The treatment by RS_CPSDMT_ interaction was statistically significant (*p* = 0.022), indicating a significant heterogeneity of treatment effect on the SDMT progression associated with different RS levels. The treatment effect on SDMT progression in potential responders and non‐responders is reported in Table 3 and in Figure 2. The treatment effect for potential responders (56% of the validation sample) was large (HR = 0.51, 95% CI: 0.27‐0.93) and significantly different from non‐responders (HR = 1.48, 95% CI: 0.78‐2.80) (interaction *p* = 0.015).

Table 4 reports variables ranked by importance and indicates that gender was the most relevant driver of response to treatment on the SDMT, favoring females.

### 3.5 Practical Application of Predictive Scores for Treatment Response

To illustrate the practical application of these scoring systems, we calculate response scores for three hypothetical patients (see also Supplementary Excel Spreadsheet).

The first patient is a 55‐year‐old woman with an EDSS of 3, who experienced five relapses in the two years before starting treatment and had a disease duration of approximately three years. Her response scores for EDSS, 9HPT, T25FW, and SDMT were ‐0.19, ‐0.11, ‐0.24, and ‐0.33, respectively. The corresponding HRs, obtained by exponentiating these scores, were 0.83, 0.89, 0.79, and 0.72. Based on predefined cut‐off values, she was classified as a non‐responder for EDSS and 9HPT but a responder for T25FW and SDMT.

The second patient is a 45‐year‐old man with an EDSS of 5, who had one relapse in the two years before treatment initiation and a disease duration of approximately four years. According to the classification criteria, he was identified as a responder for EDSS and 9HPT but a non‐responder for T25FW and SDMT.

Finally, the third patient is a 30‐year‐old woman with an EDSS of 2, who experienced six relapses in two years and had a disease duration of 2 years. Her response scores for EDSS, 9HPT, T25FW, and SDMT were ‐0.47, ‐0.24, ‐0.66, and ‐0.40, resulting in HRs of 0.63, 0.79, 0.52, and 0.67. Based on these values, she was classified as a responder for all four outcomes.

### 3.6 Relationships between the Response Scores

Table 5 shows correlations between the RS estimated for the four outcomes. The EDSS potential responders have a baseline profile well correlated to the 9HPT (r = 0.73) and T25FW (r = 0.72) responders, but not with the SDMT (r = 0.14). The 9HPT responders have low correlation with the T25FW (r = 0.29) responders and seem to be very different to the SDMT responders with a low and negative correlation (r = ‐0.11).

**TABLE 5:** Correlations between scores on the validation set (N = 493).

	RS_EDSS	RS_9HPT	RS_T25FW	RS_SDMT
RS_EDSS	**1.00**			
RS_9HPT	**0.73**	**1.00**		
RS_T25FW	**0.72**	0.29	**1.00**	0.48
RS_SDMT	0.14	−0.11	**0.48**	**1.00**

Abbreviations: 9HPT: 9‐Hole Peg Test;EDSS: expanded disability status scale; RS = response Score; SDMT: symbol digit modalities test; T25FW: timed 25‐foot walk test

### 3.7 Evaluation of the Responses Score in the Full EXPAND Dataset

Finally, (Supplementary Appendix‐B) reports all the RS generated for each outcome on the full EXPAND dataset to facilitate future validation studies.

### 3.8 Response Scores with Additional Baseline Variables

The (Supplementary Appendix‐B (Supplementary Material Table S2)) details all RS generated by the CLINICAL, ADVANCED CLINICAL, MRI, and EXPERIMENTAL set of baseline variables. The inclusion of additional baseline variables enhances the capability to distinguish between responders and non‐responders. However, this expansion comes with the cost of a reduced replicability rate of the selected RS model. Regarding EDSS progression, the inclusion of additional variables in the CLINICAL set provides minimal improvement. The AUC decreases from 0.40 with three clinical variables to 0.30 with eight variables. Moreover, replicability decreases from 71% to 57%. In contrast, the ability to discriminate between responders and non‐responders improves with the additional experimental variables for the 9HPT and T25FW progression. For the 9HPT, the AUC for the CLINICAL set is 0.59 with just one variable but decreases to 0.20 when MRI variables are included. This results in a decrease in replicability from 73% to 67%. Similarly, for the T25FW, the AUC for the CLINICAL set (four variables) is 0.49, decreasing to 0.28 for the eight‐variable EXPERIMENTAL set. However, the replicability remains at 75%. Finally, in the case of responders on SDMT progression, it appears that the addition of GFAP and the T2 lesion volume improves the ability of the RS to identify responders to Siponimod (Supplementary Material Table S3). The AUC decreases from 0.58 for the CLINICAL set (with a replicability of 76%) to 0.38 for the EXPERIMENTAL set (with a replicability of 71%).

## 4 Discussion

The importance of identifying patients with optimal suitability to available treatment options is significant but remains challenging in RRMS. With several approved drugs now available, treatment response‐specific biomarkers remain sparse, and consequently, guidance towards the most suitable treatment options is lacking. Determining the characteristics of patients with a responder profile is especially important in the progressive phase of MS, where treatment benefits can be elusive in some cases. The identification of individuals most likely to respond positively to treatments offers the potential to optimize therapeutic strategies and improve treatment outcomes in the active phase of the disease.

Progression assessment is particularly challenging in MS, and treatment effect is further impacted where progression outcomes used in clinical trials are centered on longitudinal EDSS assessment to meet regulatory requirements. Clinical trials of progressive MS (Montalban et al. 2017; Kappos et al. 2018) that, on average, demonstrate treatment benefit help some patients but not all. A major focus of personalized medicine is to better understand HTEs. The multi‐dimensional nature of MS disease progression and the heterogeneity across patient profiles lead to diverse patient responses, potentially masking the specificity of effect when aggregated into the average treatment effect most often reported. This approach has already been applied in MS studies (Pellegrini et al. 2020; Bovis et al. 2019, 2021, 2019, 2021; Falet et al. 2022), but the novelty of this analysis lies in identifying responders based on multiple outcomes, following the assumption that different patients may benefit in different domains.

This analysis demonstrated the ability to identify subgroups of patients with more pronounced Siponimod treatment benefits on different outcomes targeting different domains and that these subgroups can be characterized by different combinations of their baseline characteristics. In light of the data‐driven nature of the outlined approach and possible limitations (highlighted below), we advise extreme caution in the interpretation of these findings prior to confirmation through additional post‐marketing study research and suitable post‐hoc analyses that are needed to validate, refine, and maybe simplify the RS for their use in clinical practice. In fact, even if the simplest version of the RS (the one based on the CLINICAL PRACTICE set of variables) is extremely easy to estimate for each MS patient in the clinical practice, its use to determine the probability of response to Siponimod needs further validation on long‐term observational datasets.

This analysis is run on four levels: using a small set of routine clinical variables, an enhanced set with additional clinical variables, a conventional MRI variable set, and finally a set enriched with advanced MRI techniques and biomarkers. These steps show:

1. Patients with large Siponimod treatment benefits were clearly differentiated from non‐responders on all considered output variables based solely on routine clinical variables (age, sex, EDSS, disease duration). The response on EDSS, 9HPT, SDMT, and T25FW is modulated by the routine variables as shown by the variable ranking importance. Potential responders on the EDSS were younger and with less disability; on the 9HPT were the younger subjects; on the T25FW were patients with a lower EDSS despite a longer disease duration; and finally, those showing a larger benefit on SDMT were female.
2. Enriching the baseline clinical variables used to build the response profile only minimally increases the ability to discriminate responders and non‐responders but limits the internal validity of the findings (i.e., the replication rate of selected RS was less frequently achieved). Therefore, response scores based on features that can be readily applied in clinical practice were prioritized.
3. Applying this novel interpretation approach to the results of a clinical trial enables us to convey a different message to clinicians and patients with MS regarding the treatment's effects. The message that Siponimod treatment leads to a 21% reduction in the rate of EDSS progression then becomes that by defining responders as those patients within the first quartile of RS, these patients undergoing Siponimod treatment have a 68% chance of experiencing a 50% reduction in the progression in at least one of the four domains (EDSS, 9HPT, T25FW, SDMT). Additionally, we can estimate the most likely domain to exhibit improvement based on an individual's baseline profile.
4. Results from these kinds of unsupervised analyses can be used in a reverse way to better elucidate the biological mechanisms behind the RS in each domain.

Highlighting the potential drawback of spurious findings in such HTE assessments is important to avoid erroneously failing to provide treatment benefit to those patients within weak and/or non‐responder subgroups.

A brief summary of limitations with the approach conducted is provided here:

The risk of overfitting, where the estimation of an RS is tailored and optimized to the specific dataset used to generate it, is high as for all the predictive HTE approaches based on treatment effect modeling methods. (Kent et al. 2018; Sormani et al. 2023) This risk is further inflated with the ‘all possible subset regression’ approach, where the multiple model/testing results in overfitting. Multiplicity adjustment using a shrinkage estimator such as the *lasso* could alleviate the overfitting.

These analyses used only the independent validation data set to assess all results and the RS performance. We split the dataset into training and validation samples to accommodate the single randomized control trial. Thus, we evaluated the model performances on 30% of the original trial's size. Data splitting and feature selection uncertainty can introduce significant variations between training and validation results. Bootstrapping and overoptimism correction techniques may mitigate this problem.

We did not include biomarker‐by‐treatment terms in our model selection and evaluation. The original trial was not designed to assess interactions with baseline variables, thus a *p*‐value of ≤ 0.20 to show a satisfactory interaction level. Testing with more relaxed thresholds can increase power but can also raise type 1 errors.

Finding a subgroup with a higher treatment effect implies the other subgroup has a lower effect. In these analyses, some subgroups appear to have a negative treatment effect, but this is due to random variations around the no treatment effect estimate. Conversely, the responders group has a clear and significant treatment effect that is different from zero. The whole dataset analysis (Supplementary Appendix‐B) gives more reliable treatment effect estimates.

The suggested adaptations and considerations to mitigate the outlined limitations could be applied in future research with replication on larger datasets warranted. Moreover, to increase our ability to personalize the use of MS therapies, we must ensure that we also capture biomarker‐based characteristics that may influence outcomes, including comorbidities and social determinants of health to be added to the standard clinical measures.

## 5 Conclusions

This analysis demonstrated the ability to define responders to a therapy based on their baseline profile and evaluate the treatment effect on multiple endpoints showing that the benefit on different outcomes can vary across patients.

## Author Contributions

**Francesca Bovis**: formal analysis, conceptualization, writing–original draft, writing–review and editing, methodology, investigation, data curation, and project administration. **Ludwig Kappos**: conceptualization, writing–review and editing, methodology, investigation. **Sophie Arnould**: formal analysis, writing–review and editing, methodology, conceptualization, funding acquisition, and investigation. **Goeril Karlsson**: conceptualization, methodology, writing–review and editing, and funding acquisition. **Maria Pia Sormani**: conceptualization, methodology, data curation, supervision, formal analysis, project administration, writing–original draft, writing–review and editing, investigation, and funding acquisition.

## Conflicts of Interest

Francesca Bovis received teaching honoraria from Novartis and has received personal compensation for consulting services from EISAI.

Ludwig Kappos has received consultancy fees from Actelion, Bayer HealthCare, Biogen, BMS, Genzyme, Janssen, Japan Tobacco, Merck, Novartis, Roche, Sanofi, Santhera, and TG therapeutics; contracted research from Bayer HealthCare, Biogen, European Union, InnoSwiss, Merck, Novartis, Roche, Swiss MS Society, and Swiss National Research Foundation; speaker fees from Bayer HealthCare, Biogen, Merck, Novartis, Roche, and Sanofi; serves on the steering committee for Actelion, Bayer HealthCare, Biogen, BMS, Genzyme, Janssen, Japan Tobacco, Merck, Novartis, Roche, Sanofi, Santhera, TG Therapeutics; Support of educational activities from Allergan, Bayer HealthCare, Biogen, CSL Behring, Desitin, Genzyme, Merck, Novartis, Roche, Pfizer, Sanofi, Shire, and Teva; License fees for Neurostatus products.

Sophie Arnould, Goeril Karlsson, and Daniela Piani Meier are employees of Novartis AG,

Maria Pia Sormani has received personal compensation for consulting services and for speaking activities from Merck, Novartis, Roche, Sanofi, Bristol‐Meyer Squibb, Immunic and Biogen.

## Peer Review

The peer review history for this article is available at https://publons.com/publon/10.1002/brb3.70459.

## Supporting information



Supporting Information

## Data Availability

The reader can request the raw data (anonymized) and related documents (e.g., protocol, reporting and analysis plan, clinical study report) that underlie the results reported in this article by connecting to https://www.clinicalstudydatarequest.com and signing a Data Sharing Agreement with Novartis. These will be made available to qualified external researchers, with requests reviewed and approved by an independent review panel on the basis of scientific merit.
