# Supplementary material for: Personalized Treatment Response in Progressive MS: Can the Patient's Profile Influence the Outcome?
Source: Brain Behav. 2025 Jun 10;15(6):e70459. doi: 10.1002/brb3.70459 (PMC12152264; doi:10.1002/brb3.70459)
Supplement: Supplementary file 1 — Supporting Information [file BRB3-15-e70459-s001.docx]

**Personalized treatment response in progressive MS: can the patient’s profile influence the outcome?**

Francesca Bovis^1^, Ludwig Kappos^2^, Sophie Arnould^3^, Goeril Karlsson^3^, Maria Pia Sormani^1,4*^

Contents

[**APPENDIX – A** 2](#_Toc185328519)

[**APPENDIX – B** 3](#_Toc185328520)

[**Supplementary sTable 1: Comparison of demographics, clinical characteristics, and outcomes between the TRAIN and VALIDATION cohorts** 3](#_Toc185328521)

[**Supplementary sTable 2**: Response Score obtained on the training set using clinical, MRI, and biomarkers subgroup of variables. The performance (replicability rate and p for treatment by RS interaction were obtained on the validation set). 4](#_Toc185328522)

[**Supplementary sTable 3.** Variables predicting treatment response score, ranked in order of importance 5](#_Toc185328523)

[**Supplementary sTable 4:** Treatment effect on progression outcomes by response group in the validation set (N=493) 7](#_Toc185328524)

[**Supplementary sTable 5:** Treatment effect on progression outcomes by response group on the whole population enrolled (N=1,645) 10](#_Toc185328525)

# **APPENDIX – A**

**Score Creation**

The score building followed the modeling approach described by Zhao et al^20^. For each subject *i*, we can observe the four values ($T_{i}$, $Y_{i}$, $A_{i}$, $Z_{i}$), where $T_{i}$ is the time to disability progression, $Y_{i}$is the progression event (yes/no), $A_{i}$ is the treatment arm (PL = placebo and SPN = siponimod), and $Z_{i}$ is the covariate vector ($Z_{1i}, Z_{2i}$,..., $Z_{pi}$) made of the values of all the baseline characteristics. By fitting two separate Cox models one for each arm, we obtain the following models:

$$\left\{ \begin{aligned} \log\left( h_{PL}\left( t \right) \right)=log(h_{0PL}\left( t \right))+(\beta_{1PL}*Z_{1}+\ldots+\beta_{pPL}*Z_{p}) \\ \log\left( h_{SPN}\left( t \right) \right)=log(h_{0SPN}\left( t \right))+(\beta_{1SPN}*Z_{1}+\ldots+\beta_{pSPN}*Z_{p}) \end{aligned} \right.$$

where $h\left( t \right)$ is the hazard function.

Assuming a common baseline hazard function $h_{0}\left( t \right)=h_{0PL}\left( t \right)=h_{0SPN}\left( t \right)$, due to the randomized nature of the two groups, it is possible to calculate a response score:

$$RS\left( Z \right)=\log\left( HR \right)=(\beta_{1SPN}-\beta_{1PL})*Z_{1}+\ldots+(\beta_{pSPN}-\beta_{pPL})*Z_{p}$$

The response score $RS\left( Z \right)$ is the patient-specific score (Z representing the set of patient-specific covariates), predicting the size of treatment effect according to the patient profile.

**Model Selection**

Here, we present a practical example of the procedure used to select the best model in the training set, using EDSS outcome and the CLINICAL PRACTICE subgroup.

Following the methodology described above, we generated 31 possible response scores (RS) for each patient in the training set, corresponding to all possible 2^5^ −1 combinations of the five baseline variables included in the model (listed in the first column of the table below).

To identify the best RS, we first classified patients into potential responders and potential non-responders. This classification was based on an arbitrary cut-off set at the first quartile of the RS distribution:

- Patients with an RS below this threshold were considered responders
- Patients with an RS above this threshold were considered non-responders

Binarizing the outcome was necessary to increase the statistical power of the analysis. This approach mitigates the risk of obtaining non-significant results despite clinically meaningful treatment effects, which can occur with limited sample sizes and a relatively small number of events.

For example, consider the RS derived from a combination of age, EDSS, gender, and disease duration:

- The hazard ratio (HR) for potential responders was 0.67
- The HR for non-responders was 0.92
- The p-value for treatment-by-score interaction was 0.118

To systematically compare models, we ranked all RS based on their ability to differentiate between responders and non-responders. This ability was quantified as the ratio of treatment effects (HR responders / HR non-responders) — a measure of how strongly the treatment effect differs between the two groups.

The model with the lowest HR ratio (indicating the greatest difference in treatment response) was selected for validation. In our example, the best-performing RS for distinguishing responders from non-responders was derived from age, EDSS, and the number of relapses in the two years before treatment initiation (Ratio=0.47).

This model was then tested in the validation set.

The RS in the validation set was considered validated if the HR ratio was below 0.80, indicating at least a 20% reduction in the risk of progression or if the p-value for treatment-by-score interaction was <0.20 (a more lenient threshold due to the low power of interaction tests).

| Model List | POTENTIAL RESPONDERS | POTENTIAL NON RESPONDERS | RATIO | p x treatment interaction |
| --- | --- | --- | --- | --- |
| ~Surv(TTEDSS3M, PROGEDSS3M)AGE + EDSS + rel2ypre | 0.52 | 1.12 | 0.47 | 0.142 |
| ~Surv(TTEDSS3M, PROGEDSS3M)AGE + EDSS + logdisdur | 0.66 | 1.39 | 0.47 | 0.179 |
| ~Surv(TTEDSS3M, PROGEDSS3M)EDSS + gender + rel2ypre | 0.62 | 1.14 | 0.55 | 0.228 |
| ~Surv(TTEDSS3M, PROGEDSS3M)AGE + EDSS + gender + rel2ypre | 0.63 | 1.13 | 0.55 | 0.132 |
| ~Surv(TTEDSS3M, PROGEDSS3M)AGE + EDSS + logdisdur + rel2ypre | 0.59 | 1.00 | 0.59 | 0.082 |
| ~Surv(TTEDSS3M, PROGEDSS3M)logdisdur + rel2ypre | 0.73 | 1.21 | 0.61 | 0.239 |
| ~Surv(TTEDSS3M, PROGEDSS3M)EDSS + gender + logdisdur | 0.61 | 0.97 | 0.63 | 0.178 |
| ~Surv(TTEDSS3M, PROGEDSS3M)EDSS + gender + logdisdur + rel2ypre | 0.68 | 1.08 | 0.63 | 0.137 |
| ~Surv(TTEDSS3M, PROGEDSS3M)AGE + logdisdur + rel2ypre | 0.71 | 1.10 | 0.65 | 0.162 |
| ~Surv(TTEDSS3M, PROGEDSS3M)AGE + logdisdur | 0.64 | 0.98 | 0.65 | 0.197 |
| ~Surv(TTEDSS3M, PROGEDSS3M)EDSS + logdisdur + rel2ypre | 0.76 | 1.14 | 0.67 | 0.197 |
| ~Surv(TTEDSS3M, PROGEDSS3M)AGE + EDSS | 0.59 | 0.86 | 0.69 | 0.255 |
| ~Surv(TTEDSS3M, PROGEDSS3M)gender + logdisdur | 0.67 | 0.96 | 0.69 | 0.217 |
| ~Surv(TTEDSS3M, PROGEDSS3M)AGE + gender + rel2ypre | 0.68 | 0.96 | 0.70 | 0.198 |
| ~Surv(TTEDSS3M, PROGEDSS3M)EDSS + gender | 0.73 | 1.03 | 0.70 | 0.330 |
| ~Surv(TTEDSS3M, PROGEDSS3M)AGE + EDSS + gender + logdisdur + rel2ypre | 0.77 | 1.09 | 0.70 | 0.094 |
| ~Surv(TTEDSS3M, PROGEDSS3M)logdisdur | 0.74 | 1.05 | 0.71 | 0.310 |
| ~Surv(TTEDSS3M, PROGEDSS3M)AGE + gender + logdisdur | 0.62 | 0.86 | 0.72 | 0.137 |
| ~Surv(TTEDSS3M, PROGEDSS3M)AGE + rel2ypre | 0.65 | 0.89 | 0.73 | 0.255 |
| ~Surv(TTEDSS3M, PROGEDSS3M)AGE + EDSS + gender + logdisdur | 0.67 | 0.92 | 0.73 | 0.118 |
| ~Surv(TTEDSS3M, PROGEDSS3M)EDSS | 0.60 | 0.82 | 0.73 | 0.416 |
| ~Surv(TTEDSS3M, PROGEDSS3M)AGE + gender + logdisdur + rel2ypre | 0.69 | 0.93 | 0.74 | 0.113 |
| ~Surv(TTEDSS3M, PROGEDSS3M)EDSS + logdisdur | 0.70 | 0.94 | 0.74 | 0.260 |
| ~Surv(TTEDSS3M, PROGEDSS3M)gender + rel2ypre | 0.70 | 0.94 | 0.74 | 0.387 |
| ~Surv(TTEDSS3M, PROGEDSS3M)AGE | 0.64 | 0.81 | 0.78 | 0.364 |
| ~Surv(TTEDSS3M, PROGEDSS3M)AGE + gender | 0.69 | 0.86 | 0.81 | 0.266 |
| ~Surv(TTEDSS3M, PROGEDSS3M)gender + logdisdur + rel2ypre | 0.71 | 0.85 | 0.83 | 0.171 |
| ~Surv(TTEDSS3M, PROGEDSS3M)AGE + EDSS + gender | 0.66 | 0.79 | 0.84 | 0.178 |
| ~Surv(TTEDSS3M, PROGEDSS3M)EDSS + rel2ypre | 0.58 | 0.69 | 0.84 | 0.284 |
| ~Surv(TTEDSS3M, PROGEDSS3M)rel2ypre | 0.87 | 0.84 | 1.04 | 0.464 |
| ~Surv(TTEDSS3M, PROGEDSS3M)gender | 1.00 | 0.74 | 1.36 | 0.627 |

**Performance evaluation**

**Measure of agreement:**

Agreement was assessed qualitatively using a calibration plot. Calibration plot was generated for the selected RS on each outcome, to compare the observed vs the expected treatment effect across groups of patients with different levels of the RS, defined according to the quartiles of the RS.

**Measure of discrimination**

Computing the RS value for each patient and ranking these values in ascending order allow to build the AD(c) curve^1-3,4^ as the average treatment effect (i.e. the siponimod vs placebo HR ratio) in the subgroup of patients with RS ≤ c, where c represents the ordered RS. The smaller the value of the Area Under the Curve (AUC), the larger the ability of the RS to discriminate patient treatment effect heterogeneity.

**Rate of replication**

We used a customized rate of replication measure, developed in the context of genome-wide association studies,^5^ to evaluate how consistently we could identify a RS that distinguishes between responders and non-responders. In simple terms, we repeated the training and validation process 500 times using different bootstrapped samples. This allowed us to assess how often the RS could successfully validate across different training/validation splits. We arbitrary set a 70% success rate as a satisfactory threshold, though we recognize that further research is needed to determine the appropriateness of this threshold and its overall relevance.

**References**

1. Pellegrini F, Copetti M, Bovis F, et al. A proof-of-concept application of a novel scoring approach for personalized medicine in multiple sclerosis. Mult Scler 2020;26:1064-1073.
2. Bovis F, Carmisciano L, Signori A, et al. Defining responders to therapies by a statistical modeling approach applied to randomized clinical trial data. BMC Medicine 2019;17:113.
3. Bovis F, Kalincik T, Lublin F, et al. Treatment Response Score to Glatiramer Acetate or Interferon Beta-1a. Neurology 2021;96:e214-e227.
4. Zhao L, Tian L, Cai T, Clagget B, Wei LJ. Effectively Selecting a Target Population for a Future Comparative Study. Journal of the American Statistical Association, 2013;108: 527-39.
5. Kang G, Liu W, Cheng C, et al. Evaluation of a two-step iterative resampling procedure for internal validation of genome-wide association studies. J Hum Genet. 2015 Dec;60(12):729-38.

# **APPENDIX – B**

## **Supplementary sTable 1: Comparison of demographics, clinical characteristics, and outcomes between the TRAIN and VALIDATION cohorts**

|  | TRAIN | VALIDATION | p | Cohen’s d values |
| --- | --- | --- | --- | --- |
| n | 1152 | 493 |  |  |
| 9HPT (mean (SD)) | 34.52 (19.06) | 33.62 (18.10) | 0.372 | 0.049 |
| Age, years (mean (SD)) | 48.14 (7.85) | 47.57 (8.30) | 0.182 | 0.071 |
| EDSS (mean (SD)) | 5.45 (1.05) | 5.36 (1.09) | 0.114 | 0.084 |
| SDMT (mean (SD)) | 38.98 (13.92) | 39.25 (13.43) | 0.715 | 0.020 |
| T25FW (mean (SD)) | 16.99 (21.06) | 16.25 (21.66) | 0.520 | 0.034 |
| Disease duration, years (mean (SD)) | 14.92 (8.21) | 14.77 (8.28) | 0.749 | 0.017 |
| NBV (mean (SD)) | 1421.56 (84.22) | 1425.15 (89.77) | 0.438 | 0.041 |
| Gd+ lesion count (mean (SD)) | 0.88 (3.42) | 0.82 (3.69) | 0.750 | 0.017 |
| Relapses in the previous 2 years (mean (SD)) | 0.69 (1.21) | 0.63 (1.14) | 0.389 | 0.047 |
| logGFAP (mean (SD)) | 5.05 (0.63) | 5.02 (0.59) | 0.351 | 0.051 |
| logNFL (mean (SD)) | 3.41 (0.54) | 3.40 (0.52) | 0.769 | 0.016 |
| TAL volume (mean (SD)) | 13.93 (1.94) | 13.93 (1.88) | 0.978 | 0.002 |
| GM volume (mean (SD)) | 522.82 (53.94) | 526.84 (56.04) | 0.171 | 0.073 |
| Male sex, n (%) | 452 (39.2) | 206 (41.8) | 0.362 | 0.052 |
| Presence of Gd+ lesion, n (%) | 272 (23.6) | 108 (21.9) | 0.492 | 0.041 |
| Log T2 volume (mean (SD)) | 9.01 (1.33) | 9.04 (1.31) | 0.762 | 0.016 |
| Log T1 volume (mean (SD)) | 7.86 (1.69) | 7.88 (1.69) | 0.770 | 0.016 |
| T1T2 ratio (mean (SD)) | 0.36 (0.16) | 0.36 (0.16) | 0.878 | 0.008 |
| 3-month CDW rate - EDSS, n (%) | 329 (28.6) | 133 (27.0) | 0.553 | 0.035 |
| 3-month CDW rate - 9HPT, n (%) | 179 (15.5) | 79 (16.0) | 0.862 | 0.013 |
| 3-month CDW rate - T25FW, n (%) | 471 (40.9) | 191 (38.7) | 0.449 | 0.044 |
| 6-month CDW rate - SDMT, n (%) | 199 (17.3) | 89 (18.1) | 0.723 | 0.020 |

EDSS: Expanded disability status scale; 9HPT: 9-hole peg test; T25FW: timed 25-foot walk test; SDMT: symbol digit modalities test; NBV: normalized brain volume; GM: gray matter; TAL: normalized thalamus; NFL: neuro-filament light; GFAP: Glial Fibrillary Acidic Protein; Cohen’s *d* values represent standardized mean or proportion differences. Absolute values of *d* > 0.10 were considered clinically meaningful

The response score (RS) was first created using only demographic and clinical baseline data routinely collected in clinical practice (CLINICAL PRACTICE subgroup). The score was then assessed within the additional clinical information on the 9HPT, the T25FW and the SDMT scores (ADVANCED CLINICAL subgroup), the Gd+ lesion count and T2 lesion volume variables (CLINICAL and MRI subgroup) and finally adding biomarkers used at research level (EXPERIMENTAL subgroup) to check whether adding potentially informative variables would significantly improve the discrimination ability of the score itself.

The RS obtained on the training set (N=1,152) using the ADVANCE CLINICAL and the CLINICAL and MRI and the EXPERIMENTAL subgroup of variables were validated in the validation set (N=493) (Supplementary Table 2). In the table also the replicability rate for each RS was presented, to assess the replication rate of the RS independently from the selection of the training/validation split.

## **Supplementary sTable 2**: Response Score obtained on the training set using clinical, MRI, and biomarkers subgroup of variables. The performance (replicability rate and p for treatment by RS interaction were obtained on the validation set).

| **Outcome** | **Replicability rate (%)**** | **Response Score*** | **P for treatment by score interaction**** | **AUC** |
| --- | --- | --- | --- | --- |
| **EDSS – ADVANCED CLINICAL SET** | **67%** | -1.11 + 0.01 x Age + 0.03 x EDSS – 0.07 x Relapse + 0.005 X 9HPT + 0.0006 x T25FW | 0.115 | 0.330 |
| **EDSS – CLINICAL AND MRI SET** | **62%** | 0.04 + 0.08 x EDSS + 0.13 x male sex – 0.03 x Relapse + 0.007 x 9HPT – 0.07 x Gd+lesions – 0.10 x log(T2 lesions volume) | 0.094 | 0.278 |
| **EDSS – EXPERIMENTAL SET** | **57%** | 2.04 + 0.06 x EDSS – 0.04 x Relapse + 0.006 X 9HPT + 0.001 x T25FW – 0.06 x Gd+lesions – 0.14 x log(T2lesions volume) – 0.002 x GM – 0.01 x TAL – 0.07 x log(GFAP) | 0.040 | 0.302 |
| **9HPT – ADVANCED CLINICAL SET** | **71%** | -0.39 + 0.005 x Age | 0.078 | 0.587 |
| **9HPT – CLINICAL AND MRI SET** | **67%** | -0.83 – 0.10 x Relapse + 0.0005 x 9HPT – 0.12 x Gd+lesions | 0.235 | 0.182 |
| **9HPT – EXPERIMENTAL SET** | **55%** | 0.38 + 0.006 x Age – 0.08 x Relapse + 0.00005 x GM volume – 0.16 x log(GFAP) | 0.072 | 0.389 |
| **T25FW – ADVANCED CLINICAL** | **62%** | -0.07+ 0.08 x EDSS + 0.09 x male sex – 0.18 x log(disease duration) – 0.07 x Relapse + 0.001 x NHPT | 0.090 | 0.398 |
| **T25FW – CLINICAL AND MRI SET** | **67%** | -0.83 + 0.01 x Age + 0.13 x EDSS + 0.17 x male sex – 0.19 x log(disease duration) – 0.09 x Relapse + 0.005 x SDMT – 0.03 x Gd+lesions | 0.009 | 0.308 |
| **T25FW – EXPERIMENTAL SET** | **75%** | 3.55 + 0.005 x Age + 0.06 x EDSS + 0.11 x male sex – 0.11 x log(disease duration) + 0.004 x 9HPT – 0.31 x log(T2volume) – 0.004 x GM + 0.21 x log(NFL) | 0.085 | 0.286 |
| **SDMT – ADVANCED CLINICAL** | **74%** | -0.79 – 0.001 x Age + 0.09 x EDSS + 0.42 x Male gender – 0.14 x log(disease duration) + 0.002 x 9HPT+ 0.005 x SDMT | 0.016 | 0.548 |
| **SDMT – CLINICAL AND MRI SET** | **70%** | -0.38 + 0.0004 x Age + 0.43 x Male gender – 0.15 x log(disease duration) – 0.07 x relapse + 0.003 x 9HPT+ 0.005 x SDMT + 0.02 x Gd+lesions | 0.008 | 0.462 |
| **SDMT – EXPERIMENTAL SET** | **71%** | 2.07 + 0.32 x Male gender – 0.15 x log(disease duration) – 0.001 x T25FW + 0.02 x Gd+lesions – 0.22 x log(T2 volume) – 0.002 x GM volume – 0.06 x TAL volume+ 0.42 x log(NFL) | 0.013 | 0.359 |

EDSS: Expanded disability status scale; 9HPT: 9-hole peg test; T25FW: timed 25-foot walk test; SDMT: symbol digit modalities test; rel2years: relapses in the previous 2 years; GM: gray matter; TAL: normalized thalamus; NFL: neuro-filament light; GFAP: Glial Fibrillary Acidic Protein; AUC: area under the AD(q) curve represents the curve generated by plotting the cumulative distribution of patients ranked by individual treatment response score and the overall treatment effect relative to a given proportion of patients. The lower is the curve, the higher the heterogeneity of treatment effect.

*obtained in the training set (N=1,152)**obtained in the validation set (N=493)

The relevance of the treatment effect modifiers included in the treatment response score was evaluated using a random forest model that predicted the treatment response score based on all its baseline components. The resulting variable importance ranking was shown in Supplementary sTable 3.

## **Supplementary sTable 3.** Variables predicting treatment response score, ranked in order of importance

|  | **Variables included in the Response Score** | **VI** | **%VI** |
| --- | --- | --- | --- |
| **EDSS**  **ADVANCED CLINICAL SET** | Age | 0.017 | 100 |
|  | 9HPT | 0.015 | 90 |
|  | No. 2-year prior relapses | 0.002 | 15 |
|  | EDSS | 0.001 | 6 |
|  | T25FW | 0.000001 | 0 |
| **EDSS**  **CLINICAL AND MRI SET** | log(T2 volume) | 0.010 | 100 |
|  | GD+ lesions | 0.008 | 79 |
|  | 9HPT | 0.007 | 67 |
|  | EDSS | 0.004 | 37 |
|  | No. 2-year prior relapses | 0.002 | 16 |
|  | Male sex | 0.0007 | 7 |
| **EDSS**  **EXPERIMENTAL SET** | GD+ lesions | 0.010 | 100 |
|  | log(T2 volume) | 0.009 | 83 |
|  | 9HPT | 0.004 | 38 |
|  | EDSS | 0.003 | 28 |
|  | GM volume | 0.003 | 26 |
|  | No. 2-year prior relapses | 0.0003 | 3 |
|  | Log(GFAP) | 0.0001 | 1 |
|  | TAL | 0.00002 | 0 |
|  | T25FW | 0.00001 | 0 |
| **9HPT**  **ADVANCED CLINICAL SET *** | Age | 0.004 | 100 |
| **9HPT**  **CLINICAL AND MRI SET** | GD+ lesions | 0.14 | 100 |
|  | No. 2-year prior relapses | 0.02 | 12 |
|  | 9HPT | 0.0001 | 0 |
| **9HPT**  **EXPERIMENTAL SET** | Log(GFAP) | 0.013 | 100 |
|  | No. 2-year prior relapses | 0.010 | 75 |
|  | Age | 0.002 | 19 |
|  | GM volume | 0.000 | 0 |
| **T25FW**  **ADVANCED CLINICAL SET** | log(disease duration) | 0.02 | 100 |
|  | EDSS | 0.008 | 44 |
|  | No. 2-year prior relapses | 0.005 | 30 |
|  | Male sex | 0.001 | 6 |
|  | 9HPT | 0.0002 | 1 |
| **T25FW**  **CLINICAL AND MRI SET** | EDSS | 0.013 | 100 |
|  | log(disease duration) | 0.007 | 51 |
|  | No. 2-year prior relapses | 0.005 | 40 |
|  | Male sex | 0.004 | 28 |
|  | SDMT | 0.003 | 21 |
|  | Gd+ lesions | 0.001 | 8 |
|  | Age | 0.0003 | 2 |
| **T25FW**  **EXPERIMENTAL SET** | Log (T2 lesions volume) | 0.057 | 100 |
|  | GM volume | 0.013 | 22 |
|  | Log (NLF) | 0.002 | 4 |
|  | 9HPT | 0.001 | 2 |
|  | EDSS | 0.001 | 2 |
|  | Log(disease duration) | 0.0004 | 1 |
|  | Male gender | 0.0001 | 0 |
|  | Age | 0.0001 | 0 |
|  |  |  |  |
| **SDMT**  **ADVANCED CLINICAL SET** | Male sex | 0.028 | 100 |
|  | Log (disease duration) | 0.010 | 35 |
|  | EDSS | 0.007 | 26 |
|  | SDMT | 0.002 | 6 |
|  | 9HPT | 0.0002 | 1 |
|  | Age | 0.0000 | 0 |
| **SDMT**  **CLINICAL AND MRI SET** | Male sex | 0.03 | 100 |
|  | Log (disease duration) | 0.01 | 40 |
|  | No. 2-year prior relapses | 0.003 | 9 |
|  | 9HPT | 0.002 | 6 |
|  | SDMT | 0.002 | 5 |
|  | Gd+ lesions | 0.0002 | 1 |
|  | Age | 0.0000 | 0 |
| **SDMT**  **EXPERIMENTAL SET** | log (NFL) | 0.033 | 100 |
|  | log (T2 volume) | 0.016 | 48 |
|  | Male gender | 0.013 | 38 |
|  | log (disease duration) | 0.003 | 10 |
|  | GM volume | 0.0008 | 2 |
|  | Tal volume | 0.0005 | 1 |
|  | GD+ lesions | 0.0004 | 1 |
|  | T25FW | 0.0000 | 0 |

*****Only variable AGE was retained in the final model and therefore the ADVANCE CLINICAL SET and the CLINICAL PRACTICE set were the same.

In Supplementary sTable 4 were reported the observed treatment effect on study outcomes in patients grouped according to those having a RS higher than the overall treatment effect on the whole population (potential non-responders) or lower than the overall treatment effect on the whole population (potential responders).

## **Supplementary sTable 4:** Treatment effect on progression outcomes by response group in the validation set (N=493)

|  | **RS cut-off** | **Score group** | **Number of patients** | | **Treatment effect** | |
| --- | --- | --- | --- | --- | --- | --- |
|  |  |  | **Placebo** | **Treatment** | **HR (95% CI)** | **p*** |
| **EDSS**  **ADVANCED CLINICAL SET** | -0.24 | Potential Responders  N=232  N=412 | 84 | 148 | **0.59 (0.36-0.97)** | 0.043 |
|  |  | Potential Non-responders  N=261  N=1233 | 77 | 184 | **1.22 (0.71-2.12)** |  |
|  |  |  |  |  |  |  |
| **EDSS**  **CLINICAL and MRI SET** | -0.24 | Potential Responders  N=222 | 72 | 150 | **0.74 (0.44-1.26)** | 0.581 |
|  |  | Potential Non-responders  N=271 | 89 | 182 | **0.90 (0.56-1.46)** |  |
|  |  |  |  |  |  |  |
| **EDSS**  **EXPERIMENTAL SET** | -0.24 | Potential Responders  N=245 | 83 | 162 | **0.68 (0.42-1.11)** | 0.232 |
|  |  | Potential Non-responders  N=248 | 78 | 170 | **1.04 (0.61-1.76)** |  |
|  |  |  |  |  |  |  |
| **9HPT**  **ADVANCED CLINICAL SET*** | -0.15 | Potential Responders  N=226  N=412 | 78 | 148 | **0.57 (0.29-1.09)** | 0.075 |
|  |  | Potential Non responders  N=267  N=1233 | 83 | 184 | **1.28 (0.64-2.54)** |  |
|  |  |  |  |  |  |  |
| **9HPT SET**  **CLINICAL and MRI SET** | -0.15 | Potential Responders  N=135 | 48 | 87 | **0.46 (0.20-1.05)** | 0.095 |
|  |  | Potential Non-responders  N=358 | 113 | 245 | **1.14 (0.64-2.04)** |  |
|  |  |  |  |  |  |  |
| **9HPT**  **EXPERIMENTAL SET** | -0.15 | Potential Responders  N=222 | 87 | 135 | **0.57 (0.30-1.11)** | 0.086 |
|  |  | Potential Non-responders  N=271 | 74 | 197 | **1.37 (0.66-2.86)** |  |
|  |  |  |  |  |  |  |
| **T25FW**  **ADVANCED CLINICAL SET** | -0.05 | Potential Responders  N=231 | 77 | 154 | **0.66 (0.42-1.05)** | 0.147 |
|  |  | Potential Non-responders  N=262 | 84 | 178 | **1.04 (0.70-1.54)** |  |
|  |  |  |  |  |  |  |
| **T25FW**  **CLINICAL and MRI SET** | -0.05 | Potential Responders  N=261 | 83 | 178 | **0.68 (0.45-1.04)** | 0.124 |
|  |  | Potential Non-responders  N=232 | 78 | 154 | **1.09 (0.71-1.65)** |  |
|  |  |  |  |  |  |  |
| **T25FW**  **EXPERIMENTAL SET** | -0.05 | Potential Responders  N=290 | 89 | 201 | **0.71 (0.47-1.07)** | 0.158 |
|  |  | Potential Non-responders  N=203 | 72 | 131 | **1.10 (0.72-1.70)** |  |
|  |  |  |  |  |  |  |
| **SDMT**  **ADVANCED CLINICAL SET** | -0.27 | Potential Responders  N=276 | 84 | 192 | **0.51 (0.28-0.92)** | 0.014 |
|  |  | Potential Non-responders  N=217 | 77 | 140 | **1.52 (0.78-2.93)** |  |
|  |  |  |  |  |  |  |
| **SDMT**  **CLINICAL and MRI SET** | -0.27 | Potential Responders  N=272 | 83 | 189 | **0.48 (0.27-0.87)** | 0.006 |
|  |  | Potential Non-responders  N=221 | 78 | 143 | **1.65 (0.83-3.27)** |  |
|  |  |  |  |  |  |  |
| **SDMT**  **EXPERIMENTAL SET** | -0.27 | Potential Responders  N=269 | 82 | 187 | **0.59 (0.31-1.09)** | 0.109 |
|  |  | Potential Non-responders  N=224 | 79 | 145 | **1.19 (0.65-2.19)** |  |

*Only variable AGE was retained in the final model and therefore the ADVANCE CLINICAL SET and the CLINICAL PRACTICE set were the same.

In Supplementary sTable 5 the RS was generated for each outcome on the full EXPAND dataset (N=1,645) to give more stable estimates of the RS coefficients to be used in future validation studies.

## **Supplementary sTable 5:** Treatment effect on progression outcomes by response group on the whole population enrolled (N=1,645)

| **OUTCOME** | **RS** | **RS cut-off** | **Score group** | **Number of patients** | | **Treatment effect** | |
| --- | --- | --- | --- | --- | --- | --- | --- |
|  |  |  |  | **Placebo** | **Treatment** | **HR (95% CI)** | **p*** |
| **EDSS** | -1.21 +0.01*Age+0.07*EDSS-0.09*2yr prior relapses | -0.24 | Potential Responders  N=715  N=412 | 251 | 464 | **0.64 (0.49-0.84)** | 0.027 |
|  |  |  | Potential Non-responders  N=930  N=1233 | 295 | 635 | **0.97 (0.74-1.27)** |  |
|  |  |  |  |  |  |  |  |
| **9HPT** | -1.05+0.02*Age | -0.15 | Potential Responders  N=668  N=412 | 217 | 451 | **0.68 (0.47-0.97)** | 0.087 |
|  |  |  | Potential Non-responders  N=977  N=1233 | 329 | 648 | **1.05 (0.73-1.50)** |  |
|  |  |  |  |  |  |  |  |
| **T25FW** | -0.61+0.01*Age+0.10*EDSS-0.21*log(disease duration)-0.09*2yr prior relapses | -0.05 | Potential Responders  N=767  N=412 | 245 | 522 | **0.77 (0.60-0.98)** | 0.017 |
|  |  |  | Potential Non-responders  N=878  N=1233 | 301 | 577 | **1.14 (0.92-1.41)** |  |
|  |  |  |  |  |  |  |  |
| **SDMT** | -0.19+0.03*EDSS-0.19*log(disease duration)+0.53*male sex | -0.27 | Potential Responders  N=928  N=412 | 306 | 622 | **0.59 (0.43-0.80)** | 0.031 |
|  |  |  | Potential Non-responders  N=717  N=1233 | 240 | 477 | **1.00 (0.69-1.44)** |  |
